# Event Related Potential Signal Capture Can Be Enhanced through Dynamic SNR-Weighted Channel Pooling

**DOI:** 10.3390/s21217258

**Published:** 2021-10-31

**Authors:** Sujoy Ghosh Hajra, Careesa C. Liu, Shaun D. Fickling, Gabriela M. Pawlowski, Xiaowei Song, Ryan C. N. D’Arcy

**Affiliations:** 1 Faculty of Applied Sciences, Simon Fraser University, Burnaby, BC V5A 1S6, Canada; sujoy.ghoshhajra@nrc.ca (S.G.H.); careesa.liu@myant.ca (C.C.L.); shaunfickling@healthtechconnex.com (S.D.F.); 2 Flight Research Laboratory, Aerospace Research Centre, National Research Council Canada, Ottawa, ON K1V 1J8, Canada; 3 Myant Inc., Toronto, ON M9W 1B6, Canada; 4 HealthTech Connex Inc., Surrey, BC V3V 0C6, Canada; gabrielapawlowski@healthtechconnex.com; 5 Faculty of Sciences, Simon Fraser University, Burnaby, BC V5A 1S6, Canada; xiaowei.song@fraserhealth.ca; 6 Health Sciences and Innovation, Surrey Memorial Hospital, Fraser Health Authority, Surrey, BC V3T 0H1, Canada; 7 DM Centre for Brain Health (Radiology), University of British Columbia, Vancouver, BC V6T 1Z3, Canada

**Keywords:** EEG, ERP, neural signal processing, signal augmentation, signal to noise ratio, channel pooling

## Abstract

Background: Electroencephalography (EEG)-derived event-related potentials (ERPs) provide information about a variety of brain functions, but often suffer from low inherent signal-to-noise ratio (SNR). To overcome the low SNR, techniques that pool data from multiple sensors have been applied. However, such pooling implicitly assumes that the SNR among sensors is equal, which is not necessarily valid. This study presents a novel approach for signal pooling that accounts for differential SNR among sensors. Methods: The new technique involves pooling together signals from multiple EEG channels weighted by their respective SNRs relative to the overall SNR of all channels. We compared ERP responses derived using this new technique with those derived using both individual channels as well as traditional averaged-based channel pooling. The outcomes were evaluated in both simulated data and real data from healthy adult volunteers (n = 37). Responses corresponding to a range of ERP components indexing auditory sensation (N100), attention (P300) and language processing (N400) were evaluated. Results: Simulation results demonstrate that, compared to traditional pooling technique, the new SNR-weighted channel pooling technique improved ERP response effect size in cases of unequal noise among channels (*p*’s < 0.001). Similarly, results from real-world experimental data showed that the new technique resulted in significantly greater ERP effect sizes compared to either traditional pooling or individual channel approach for all three ERP components (*p*’s < 0.001). Furthermore, the new channel pooling approach also resulted in larger ERP signal amplitudes as well as greater differences among experimental conditions (*p*’s < 0.001). Conclusion: These results suggest that the new technique improves the capture of ERP responses relative to traditional techniques. As such, SNR-weighted channel pooling can further enable widespread applications of ERP techniques, especially those that require rapid assessments in noisy out-of-laboratory environments.

## 1. Introduction

In recent years, advances in portable electroencephalography (EEG) technology have increasingly enabled the development of point-of-care, objective, physiology-based measurements of brain function, such as the brain vital sign monitoring [1,2,3]. Event-related potentials (ERPs) extracted from EEG can provide physiology-based measures to augment existing behaviour-based assessments of brain function, which had been shown to be highly subjective and potentially error-prone [4,5,6]. ERPs have been shown to index both low-level sensory and high-level cognitive processes [7], and both our group and others have previously demonstrated the significant utility of ERPs in capturing cognitive changes in brain function during aging [1,8], and due to brain injury, disease, and recovery [9,10,11].

**Figure 1:**
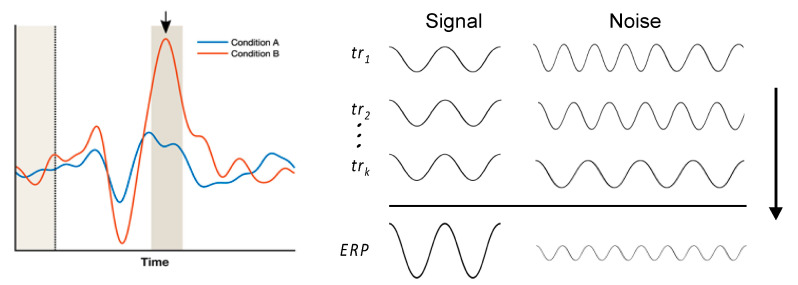
Sample ERP waveforms illustrating experimental methodology and trial averaging process. (**Left**) Brain responses to specific stimuli are measured by contrasting a target experimental condition (Condition B, orange) with a control condition (Condition A, blue). The black arrow indicates the response peak of interest, or ERP component. The black dotted line denotes stimulus onset. ERP components are typically evaluated by quantifying amplitude differences between the two conditions over an interval of interest spanning the peak (dark shaded region). Additional comparisons are also made with the signals during a pre-stimulus baseline interval (light shaded region). (**Right**) ERP waveforms are generated through conditional averaging of several trials (“tr”), each consisting of signal and noise components. This process relies on the event-related neurophysiological signal of interest (“Signal”) having relatively similar morphology and latency in each trial, in contrast to the noise components (“Noise”) being relatively dissimilar from trial to trial, leading to signal-to-noise increases by a factor of √ k.

ERP assessments in both research and clinical settings rely on comparing the brain’s responses to different types of stimuli or experimental conditions (Figure 1). ERP responses, or components, are typically identified using established features in the waveform (e.g., a peak with a specific polarity within an expected time interval [12]) that are contrasted among experimental conditions (e.g., different types of stimuli). Thus, the goal of many ERP experiments is to increase ERP response capture for improved detection of experimental effects. The amount of response captured can be quantified through effect size (ES) measurements, and can be improved by increasing the difference between the means of the experimental conditions, or by decreasing the variance of that difference. However, increasing the difference among the means is generally considered infeasible, and so reduction of variance is commonly pursued through various signal-processing approaches [13].

A common approach to reduce the variance and enhance the signal-to-noise ratio (SNR) of ERP responses is by averaging over a large number of repeated trials (Figure 1). Trial-averaging enables effective removal of non-stimulus locked EEG signals while retaining the stimulus-locked response of interest [7,14]. However, a significant drawback to this approach is the lengthy experimental time necessary for obtaining a large number of trials. Large trial numbers (with hundreds of trials being routine)—and therefore long acquisition times—are not feasible in many cases (e.g., realistic out-of-laboratory assessments) where ERP-based assessments may be utilized. Moreover, the use of long test times is further constrained by practical considerations, such as habituation, participant compliance or fatigue, as well as clinical challenges such as rapidly changing attention and awareness levels in patients [15,16].

Given the limitations on simply increasing the number of trials, an alternate approach for enhancing the signals is to combine data from several electrodes [13,17]. Pooling together data from multiple electrodes enables the effective total number of trials to be further increased (with corresponding decrease in variance), without lengthening the data collection time. However, given that the relative amounts of signal and noise content in individual channels are dependent on factors such as their respective scalp-electrode impedances [18], pooling together data from electrodes without accounting for these differences can lead to inaccuracies. Moreover, these issues are exacerbated in modern ERP experiments, which are often performed with scalp-electrode impedances in a much larger range (e.g., 0–20 kOhms) compared to traditional ERP studies (e.g., 0–5 kOhms), thereby greatly increasing the possibility of mismatched signal quality across sensors. Indeed, there have been previous reports of the blurring of the ERP response following channel pooling, resulting in attenuation rather than improvement when data from electrodes were pooled without accounting for the signal quality differences [19].

A new technique is needed that can retain the attractive features of channel pooling and enhance ERP signals without introducing inaccuracies due to cross-channel signal quality variations. In this paper, we present a new dynamic SNR-weighted (dSNRw) signal capture approach that enables the pooling of multiple channels while accommodating potential differences in signal quality among them. Specifically, our technique first quantified the relative signal-to-noise content of each channel and condition, then utilized this information to inform the combination of data across electrodes.

In this study, we demonstrated the dSNRw approach using both simulated and empirical EEG data, and hypothesized that the dSNRw technique would achieve significant improvements in capturing ERP responses compared to traditional channel pooling. Our results confirmed our hypothesis, and validated that the dSNRw technique indeed improves capture of ERP responses in both simulated data as well as in real world EEG data—with the improvements observed across a variety of established ERP components ranging from sensation (N100 ERP [20]) to attention (P300 ERP [21]) to language processing (N400 ERP [22]). Thus, by boosting the ERP response capture without requiring additional trials, the dSNRw approach, may enable more widespread application of ERP technology in realistic, out-of-the-laboratory situations, especially those that require rapid assessment.

## 2. Methods

### 2.1. Study I: Simulated Data

The primary motivation for the development of the new signal combination technique described herein was to fuse signals from multiple sensors while accounting for potential differences in SNR among the sensors. Accordingly, the first study focused on evaluating the dSNRw technique using synthetic data with well controlled parameters.

#### 2.1.1. Data Generation

**Figure 2:**
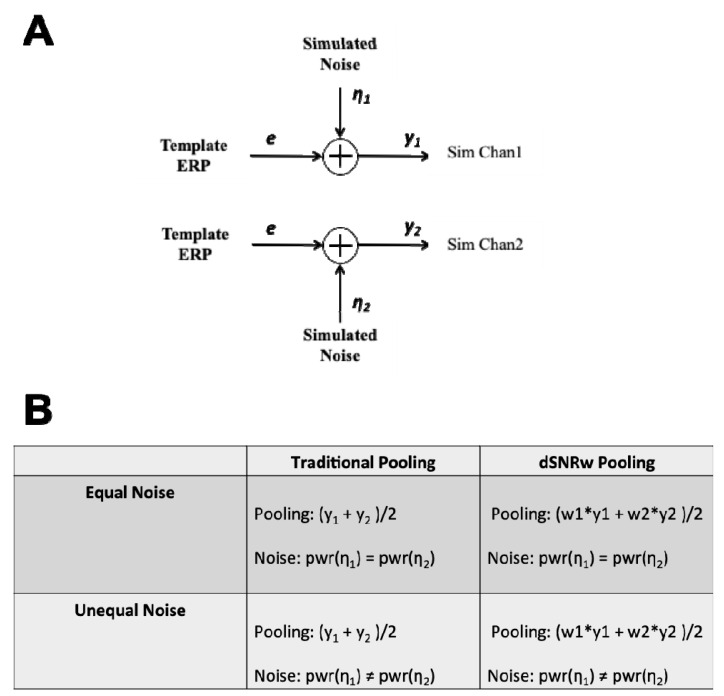
Overview of simulation process. (**A**) Template ERP waveforms derived from healthy participants were combined with simulated noise signals to create channels of simulated ERP data. (**B**) Simulated channels of data were generated under two scenarios—(1) with the power of the noise being equal in both simulated channels, and (2) power of the noise being unequal in the two simulated channels, and data from both scenarios were combined using the traditional channel pooling and the dSNRw techniques.

Simulated ERP trials were generated by combining template ERP time series vectors with simulated noise vectors as shown in Figure 2A, in line with the additive model of ERP generation [23]. The template ERP vectors representing the signal of interest were derived using data from healthy adults collected in prior works [1,2]. Specifically, the template ERP waveforms, derived from the previous studies, corresponded to the grand-averaged responses to two types of stimuli—in particular, the *standard* and *deviant* tone waveforms represented the P300 ERP response, and the *congruent* and *incongruent* language waveforms represented the N400 ERP response. Templates corresponding to both the P300 and the N400 ERP responses were chosen since they represent differing underlying neural responses. Moreover, these ERPs also have inherently different signal characteristics in that the P300 response is a large positive deflection, whereas the N400 is a relatively smaller negative deflection in the time series data. The noise vectors were generated by using a 3rd-order auto-regressive process, as shown in Equation (1), with coefficients estimated by the Burg method based on background EEG in line with prior works [24,25].

(1) $$\eta \left(t\right)=\alpha_{1}\left(t-1\right)+\alpha_{2}\left(t-2\right)+\alpha_{3}\left(t-3\right)+r\left(t\right)$$

where *η(t)* is the auto-regressive process, *r(t)* is Gaussian white noise driving the system, and parameters ($\alpha_{1},\;\alpha_{2},\;\alpha_{3}$) are estimated as described above.

The data generation process described above was repeated 100 times to simulate the generation of ERP responses from 100 individuals, all having the same signal of interest (i.e., the template ERP) but different noise vectors (i.e., the simulated additive noise). This procedure produced a single channel of simulated ERP (*Sim Chan 1*) for all 100 individuals. To simulate equal and unequal noise scenarios between different sensors, a second channel of simulated ERP (*Sim Chan 2*) was also generated using the same procedure according to two different conditions: (1) *Equal Noise:* the power of the noise vector of the second channel is equal to that of the first channel; and (2) *Unequal Noise:* the power of the noise vector for the second channel is not equal to the power of the noise vector for the first channel, and is indeed double that of the first channel (Figure 2B).

#### 2.1.2. Channel Data Fusion

Following the generation of simulated data as described above, the two channels of simulated data under both equal and unequal noise scenarios were combined using two different techniques—(a) traditional channel pooling, and (b) dSNRw channel pooling.

#### 2.1.3. Traditional Channel Pooling

The traditional technique for channel pooling entails signal augmentation through the process of averaging across the channels being pooled at each time point and for each stimulus/experimental condition, described mathematically in Equation (2):

(2) $$C_{pooled}\left(s,t\right)=\sum_{j=1}^{J}\frac{1}{J}C_{j}\left(s,t\right)$$

where *C_pooled_* is the combined signal from each channel *C_n_* for stimulus type *s* and all-time *t* within an epoch/trial, and *J* is the total number of channels being pooled.

#### 2.1.4. dSNRw Channel Pooling

The dSNRw technique performs a weighted combination of signals from the channels being pooled together, depending on the relative signal-to-noise ratios of each channel as shown in Equation (3):

(3) $$C_{dSNRw}\left(s,t\right)=\sum_{j=1}^{J}w_{j}C_{j}\left(s,t\right)$$

where *C_dSNRw_* is the combined signal from each channel *C_j_* for stimulus type *s* and all time *t* within an epoch/trial, *w_j_* is calculated as the ratio of the SNR of channel *j* divided by the sum of the SNR values of all channels in the pool (i.e., SNRj/(SNR_1_ + SNR_2_ + … + SNR_J_)), and *J* is the total number of channels pooled.

The pre-stimulus interval is not deemed to contain significant neural response of interest (signal), and therefore is used as a surrogate for the amount of noise remaining after signal-processing steps have been undertaken [26]. In contrast, the signal of interest is generally evaluated within a time interval of interest post stimulus presentation, and therefore SNR is commonly defined as shown in Equation (4):

(4) $$\mathrm{SNR}=\frac{\bar{y_{tw}}}{\sigma_{M,b}}$$

where, $\bar{y_{tw}}$ is the mean amplitude of the ERP waveform computed within a post-stimulus time interval of interest *tw* for each trial before averaging across trials, and $\sigma_{M,b}$ is the standard error across trials of the mean waveform amplitude computed within the pre-stimulus baseline internal.

#### 2.1.5. ERP Response Quantification & Comparison of Pooling Techniques

The goal of ERP experiments is to elicit differential responses to experimental conditions. The separation between experimental conditions within specific time windows of interest can be quantified using effect size (ES) measurements as shown in Equation (5):

(5) $$ES=\frac{\left|X_{iB}\left(t_{W}\right)-X_{iC}\left(t_{W}\right)\right|}{\sigma \left(t_{W}\right)}$$

where, *X_iB_* and *X_iC_* are means of conditions B and C, respectively (e.g., *deviant* and *standard* for P300, or *congruent* and *incongruent* for N400), and σ is the pooled standard deviation. Both are measured within time intervals of interest *t_W_*.

In order to compare the two channel techniques (traditional vs. dSNRw), the ES metric was used as an outcome variable within a Monte-Carlo framework. In particular, this entailed selecting a subset of 10 simulated participants (out of the 100 simulated participants), and calculating the effect size within a time interval of interest across the pairs of waveforms/conditions using both the traditional and the dSNRw techniques for each participant. The time interval of interest for ES calculations were chosen to be 250–550 ms post-stimulus for simulated P300, and 300–600 ms for simulated N400, in line with prior literature [2,27]. This process was repeated 1000 times, and paired *t*-tests were utilized to compare the outcomes of the two channel pooling techniques, with *p* < 0.001 considered to be significant. This comparison was made separately for the equal and unequal noise scenarios.

### 2.2. Study II: Experimental Data

#### 2.2.1. Participant Details

Thirty-seven healthy individuals (age 34 ± 12, 16 female) volunteered for the study. All were fluent in English, had normal hearing, and normal or corrected-to-normal vision. None had history of neurological disease or psychoactive medications. The study was approved by the Research Ethics Boards at Simon Fraser University and Fraser Health Authority, and all participants provided written informed consent prior to data acquisition.

#### 2.2.2. Stimulus Paradigm

The ERP experimental paradigm used auditory stimulation of the brain vital signs framework, which enabled the elicitation and evaluation of a spectrum of ERP brain responses including N100 [20], P300 [21] and N400 [22] in about 5 min [1]. Details of the stimulus sequences have been presented elsewhere [1,16,28]. Briefly, the tones were 250 ms in duration and were comprised of two types of sounds: a more frequently occurring *standard* tone (740 Hz, 75 dB, 80% occurrence), and a less frequently occurring *deviant* tone (1047 Hz, 100 dB, 20% occurrence). The words were presented in pairs across two equally likely experimental conditions: the *congruent* condition consisted of two words that were congruent in meaning (e.g., ‘Bread’ ‘Butter’, 50% occurrence), and an *incongruent* condition in which the words did not agree in meaning (e.g., ‘Bread’ ‘Cat’, 50% occurrence). The N100 and P300 responses were derived by comparing the standard and deviant conditions, while the N400 response was derived by contrasting the congruent and incongruent conditions.

#### 2.2.3. Data Acquisition

EEG data were collected using a 64-channel system with active Ag/Ag-Cl electrodes (BrainAmp 64-channel with actiCap) in a dedicated EEG room with consistent conditions such as lighting levels. Skin-electrode impedances were maintained below 20 kOhms, and binaural auditory stimulation was provided to the participants via insert earphones (ER4), while they maintained visual fixation on a cross, displayed on a screen 75 cm away. Stimulus delivery and time stamping signals were controlled by Presentation software (version 18.0, Neurobehavioral Systems Inc., Berkeley, CA, USA), and EEG data (with concurrent time stamping signal recording) were recorded using BrainVision Recorder software (version 1.20.0801, Brain Products GmbH, Munich, Germany).

#### 2.2.4. Data Pre-Processing and ERP Generation

Raw EEG data were first visually inspected, and artifactual channels were removed. Data were then notch-filtered to remove power line noise (60 Hz), band-pass filtered (0.1–50 Hz), and automatic artifact rejection was applied using a gradient analysis approach with 10 μV/ms as threshold. Thereafter, independent component analysis (ICA, [29,30]) was employed to select and remove artefacts corresponding to ocular, muscular, cardiac, and other sources based on previously established criteria [31]. For each channel, the ICA-cleaned data were then low-pass filtered to 20 Hz, segmented (−100 to 900 ms epochs relative to stimulus onset), baseline corrected (−100 to 0 ms), and conditionally averaged to generate the ERPs using established procedures [7]. All analyses were conducted using BrainVision Analyzer software (version 2.03, Brain Products GmbH, Munich, Germany) and custom MATLAB scripts.

#### 2.2.5. Channel Data Fusion

As described in the Study I section, the per-channel ERPs generated using the processing steps above were combined using both the traditional channel pooling (described in Equation (2)) and dSNRw channel pooling (described in Equation (3)) techniques. A major motivation for this work was to enable ERP-based approaches in realistic non-laboratory settings, and thus the focus was on utilizing minimal numbers of electrodes rather than the full-head electrode array of 64-channels. Accordingly, in line with the ERP-based rapid assessment brain vital signs technology, the analysis focused on the three midline electrodes (Fz, Cz and Pz). Specific channels to be pooled were selected based on the ERP of interest due to the spatial distribution differences among the N100, P300 and N400 ERP responses. In particular, to maximize the captured effects, Fz and Cz were pooled for N100 and P300 ERP responses give the frontal-central distribution of this ERP response, whereas Cz and Pz were pooled for N400 given the centro-parietal maxima of this response [2,16,17].

#### 2.2.6. ERP Response Quantification & Comparison of Pooling Techniques

Similar to Study I, effect size (described in Equation (5)) was used to quantify the amount of ERP response captured. In line with prior works [2], the following time windows were used for the ES calculations: 80–180 ms for N100, 250–550 ms for P300, 300–600 ms for N400. In order to compare the two channel pooling techniques, statistical analysis utilized a bootstrapping approach using sub-selection of participants from the available data pool to compare the two combinatorial techniques. Specifically, this entailed selecting a subset of 10 participants, and calculating the ES for each of the pooled signals formed using the traditional and dSNRw channel pooling techniques. This process was repeated 1000 times, and the calculated ES measures across the two pooling approaches were tabulated and stored for further analysis. Additionally, the ES measurement was also undertaken for each of the electrodes being pooled together, and the largest ES value was stored for further analysis.

One-way ANOVA was utilized to identify omnibus effects in comparing ES of differing pooling types (i.e., *single largest electrode ES*, *traditional pooling ES* and *dSNRw pooling ES*), with Bonferroni correction for post-hoc multiple comparisons. For additional comparison, ES measures derived using both the traditional and dSNRw pooling techniques were also converted into a percentage differential relative to that of the single electrode exhibiting the largest ES, and paired *t*-tests were applied to assess differences across the techniques.

In addition, qualitative visual comparison of the ERP responses derived using the traditional and dSNRw combination approaches were undertaken. Furthermore, the mean signal amplitude of the ERP responses in time windows (50 ms for N100, 100 ms for P300 and 200 ms for N400) surrounding the peaks of interest were quantified. Additionally, the difference in mean signal amplitude among the experimental conditions (i.e., standard and deviant for N100 and P300, and congruent and incongruent for N400) in the same time windows were also recorded. This process was repeated 1000 times as described above, and both the mean signal amplitude and the difference of signal amplitude among experimental conditions derived from ERPs generated using traditional and dSNRw weighted combinations were compared using paired *t*-tests. As described in the introduction, ERPs are often evaluated in terms of the relative changes among experimental conditions, and therefore the percentage change in the differences among experimental conditions due to the dSNRw combination relative to the traditional combination were also quantified.

#### 2.2.7. Supplementary Analysis

While not the primary objective of the current study, additional analysis was undertaken to assess the performance of the dSNRw technique when applied to electrodes not located along the midline. In recent years, there has been increasing interest in obtaining ERP-based metrics from in or around the ears as it provides an easy access point for application of EEG sensors [32]. In order to further assess the robustness of the dSNRw approach, the technique was also applied to combining data from sensors located near the ears (i.e., at mastoid locations). Specifically, data from T7 and T9 sensors were combined using the traditional pooling and the dSNRw pooling techniques, and compared using paired *t*-tests for assessing the impact on ES of N100, P300 and N400 ERPs.

## 3. Results

### 3.1. Study I: Simulated Data

**Figure 3:**
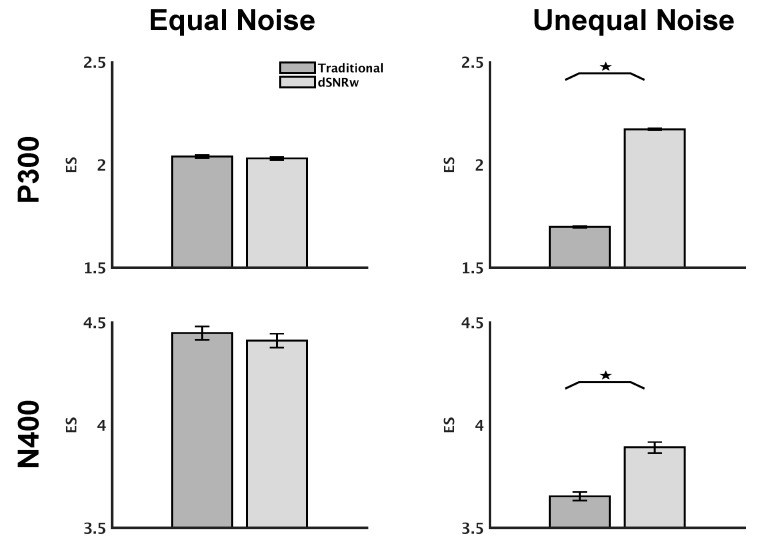
Effect size measurements for simulated P300 and N400 ERPs with varying noise levels for channels being combined. Results are presented as mean ± SEM. The dSNRw signal combinatorial approach outperformed the traditional channel pooling technique in the presence of unequal noise levels for both simulated P300 and N400 ERPs. No significant differences among the techniques were observed for the situation of equal noise levels in the channels being pooled. * *p* < 0.001 across signal pooling techniques.

Effect size measurements on simulation data showed that the dSNRw combinatorial technique better captured the difference between the ERP waveform pairs compared to traditional channel pooling (Figure 3). The improvement in signal capture provided by the dSNRw technique over the traditional pooling approach was significant when the channels being combined had varying noise levels (*p* < 0.001), but no significant differences were observed when the same level of noise was present in the channels being pooled. In fact, the effect size measures for the equal noise scenario were highly correlated across the two combination approaches (simulated P300: r = 0.8996, *p* < 0.001; simulated N400: r = 0.8792, *p* < 0.001).

### 3.2. Study II: Experimental Data

**Figure 4:**
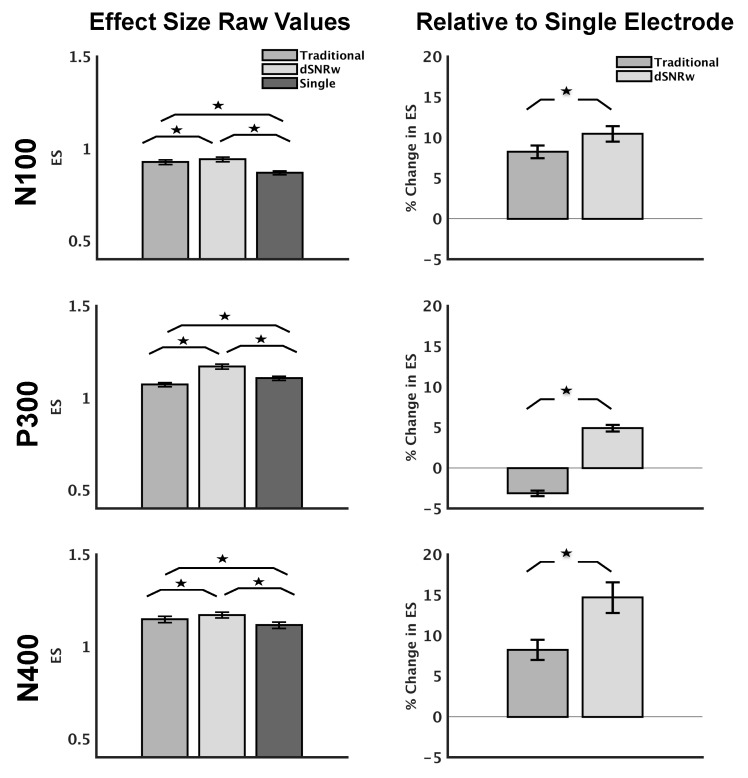
Effect size measurements for experimental N100, P300 and N400 ERPs, presented mean ± SEM. (**Left**): ES measurements for all three ERPs are shown at midline electrodes. (**Right**): The relative percentage change in ES for each of the two combinatorial approaches relative to the single electrode with the largest ES. * *p* < 0.001. Traditional, dSNRw = channel pooling techniques; Single = electrode with the largest effect size measurement.

In line with results from simulation data, the dSNRw approach also improved the measurability (as quantified by effect size) of ERPs corresponding to sensation (N100), attention (P300) and language processing (N400) in experimental data as shown in Figure 4 (left panel). The percentage change in the effect size from dSNRw and traditional channel pooling compared to that of the largest single channel ERP results are also shown in Figure 4 (right panel). Statistical analysis showed a significant effect of combination technique for N100 [F (1.4, 1376.6) = 80.7, *p* < 0.001], P300 [F (1.2, 1204.0) = 497.8, *p* < 0.001] and N400 [F (1.5, 1531.2) = 31.9, *p* < 0.001]. Post-hoc testing demonstrated that the dSNRw technique produced higher ES measurements compared to the traditional channel pooling, as well as the electrode with the largest effect size measurement (*p* < 0.001, Bonferroni corrected). The improved measurability of the ERPs is further confirmed in Figure 4 (right panel), which showed greater increase in effect size relative to the single electrode setup compared to traditional channel pooling (*p* < 0.001).

As shown in the supplementary material (Figure S1), the general pattern of dSNRw outperforming traditional channel pooling holds true for all three ERPs when applied to the signals from the mastoid channels (*p* < 0.05). However, the absolute effect size values were found to be lower for N400 in both pooling techniques. This may be a reflection of the relative proximity between the source of the N400 (left temporal lobe largest contributor) and the recording (T7, TP7) and reference (TP9) electrodes within this specific measurement scheme.

**Figure 5:**
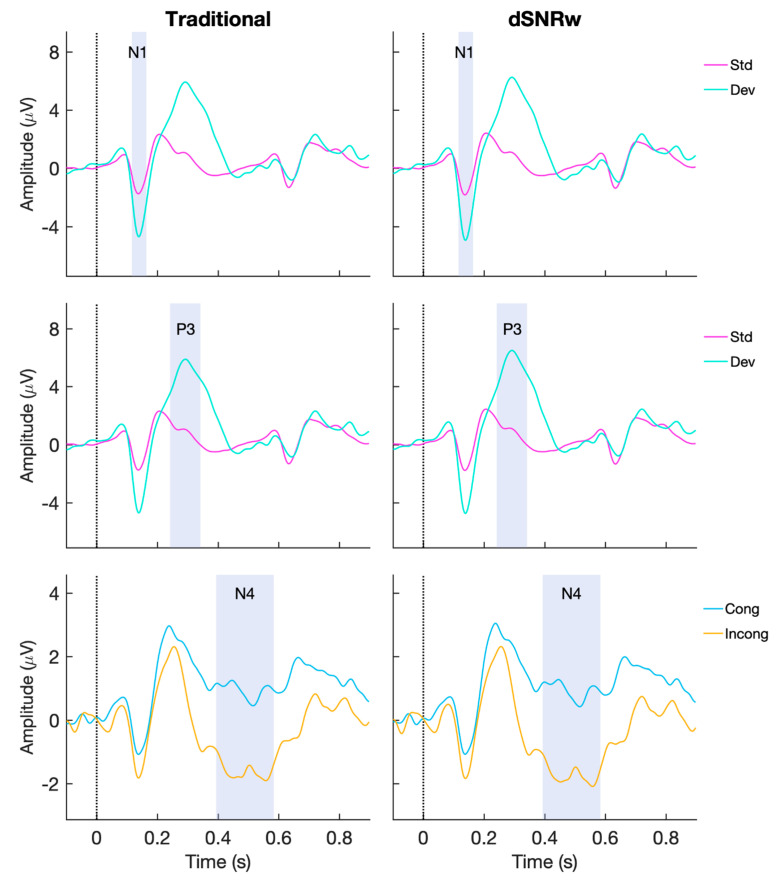
Group averaged ERP waveforms. (**Left**): ERP responses corresponding to N100 (N1), P300 (P3) and N400 (N4) derived from traditional channel pooling. (**Right**): ERP responses derived using dSNRw channel pooling. Shaded sections correspond to time windows of interest for respective ERP components. Std = standard tonal stimuli, Dev = deviant tonal stimuli, Cong = congruent word stimuli, Incong = incongruent word stimuli.

**Figure 6:**
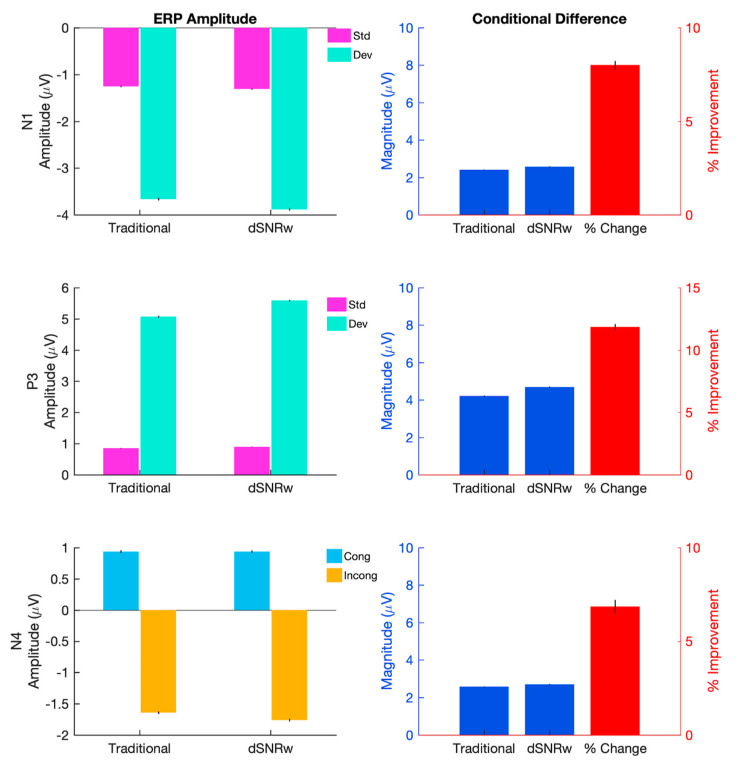
ERP amplitude measurements for N100, P300 and N400 ERPs, presented mean ± SEM. Left: ERP signal amplitude in time windows of interest, shown as shaded intervals in previous figure, for ERPs derived from traditional and dSNRw weighted pooling schemes. All pairwise *p*’s < 0.001. Right: The magnitude of the difference among experimental conditions for each channel pooling technique, as well as the relative percentage change. Pairwise comparisons of magnitude differences for each ERP: *p* < 0.001. Traditional, dSNRw = channel pooling techniques; Std = standard tonal stimuli, Dev = deviant tonal stimuli, Cong = congruent word stimuli, Incong = incongruent word stimuli; N1 = N100 ERP, P3 = P300 ERP, N4 = N400 ERP.

In addition to the above results, qualitative visual comparisons of the ERP waveforms generated by the traditional and dSNRw pooling approaches (Figure 5) reveals the similarity of the morphology of the ERP response time courses between the two approaches. In general, both techniques are able to capture the three ERP responses of interest, but the response size is larger when ERPs are generated with the dSNRw approach. Further to the qualitative assessments, quantitative comparisons (Figure 6) also confirm the superiority of the dSNRw approach. Specifically, larger signal amplitudes were captured when ERPs were generated using the dSNRw techniques compared to the traditional channel pooling approach as confirmed by pairwise comparison of experimental conditions for each the three ERPs (all *p*’s < 0.001). Similarly, comparisons of the magnitude of the differences among experimental conditions (e.g., deviant—standard condition amplitudes for P300) revealed larger differences when ERPs were generated using the dSNRw technique (*p*’s < 0.001). Indeed, these differences were up to 12% more when the dSNRw approach was utilized (Figure 6).

## 4. Discussion

### 4.1. Main Findings

In this paper, we developed and validated a novel signal pooling technique (dSNRw) that combined data from multiple electrodes/channels while accounting for the relative contributions of signal and noise within each channel. In support of our hypothesis, our results indicate that the dSNRw technique enables improved capture of ERP responses compared to traditional channel pooling as well as non-pooled individual-channel ERP responses. The effect size improvement achieved with dSNRw is demonstrated using both “ground truth” simulated data and empirical data from healthy adults for ERPs spanning the entire information processing spectrum from low-level auditory sensation (N100) to attention (P300) to high-level language processing (N400).

### 4.2. Simulated Data

Typical models of EEG generation assume the brain tissue to be resistive, and volume conduction mediated by a propagation vector with minimal capacitive effects is considered to give rise to the scalp-recorded potentials [33]. Within the context of ERP studies, the task-relevant ERPs are considered signals of interest, while all other neural activity as well as interfering non-neural artifacts are considered noise, with the superposition of signal and noise recorded at the electrodes [34]. The relative amounts of signal and noise recorded at each electrode (even adjacent ones) can therefore vary, with empirical studies having previously demonstrated the impact of various external factors such as skin-electrode impedance on the signal-to-noise ratio [18,35]. In the present study, the results from simulated data demonstrate that, when equal background noise is present in the channels being combined, both traditional channel pooling as well as the dSNRw technique capture similar levels of ERP effects (Figure 3 left panel). However, in the more realistic scenario of unequal noise levels among channels, the proposed dSNRw technique far outperforms the traditional channel pooling approach (Figure 3 right panel).

These results provide the foundational ‘ground truth’ verification of our primary hypothesis that a technique that accounts for SNR differences among channels would better capture ERP effects compared to the traditional channel pooling technique, which simply averages signals across channels without accounting for signal quality differences. Analytically, the impact of channel pooling on the noise portion can be modelled as a mixture of Gaussian distributions; and since the noise terms of each channel being pooled is considered to be zero-mean due to signal processing steps, the noise mixture when pooling channels can also be considered a weighted mixture of the underlying variances of the noise terms. Specifically, this means that, in the case of traditional channel pooling, the amount of variance introduced due to the noise term is simply a mean of the variances of the noise terms of each of the constituent channels in the pool. If the component channel noise variances are equal, the variance of the noise component of the pooled channels remains the same, and the measurability of the ERP effect improves due to the impact of trial averaging as previously mentioned. However, if the two variances are unequal (e.g., variance of one channel is double that of another), the variance of the pool actually becomes larger than the original variance of each channel, thereby increasing the impact of the noise and potentially obscuring the ERP effect. However, weighting the constituent channels of the pool differently based on the relative SNR, rather than a constant weighting as is applied in the traditional channel pooling technique, can mitigate the negative impact of the mismatched variances. Indeed, our simulation results confirm this analytical view.

### 4.3. Experimental Data

Following the successful demonstration of the proposed technique using simulated data, the same technique was applied to real world experimental data collected using the brain vital signs framework [1] capturing neural responses corresponding to sensation (N100), attention (P300) and language processing (N400). Improvements in the measurement of ERP responses were observed for all three neural markers using the dSNRw technique relative to both the single best electrode/channel as well as the traditional channel pooling technique (Figure 4).

For the P300 response, the traditional channel pooling technique resulted in a reduction of effect size, and may be reflective of prior reports suggesting the blurring of amplitude effects when traditional channel pooling is applied [13,19]. In contrast, the application of the dSNRw technique resulted in an increase in the measured effect size of the P300 response. Similarly, the dSNRw technique resulted in a near-doubling of effect size of the N400 response (8% improvement with traditional pooling vs. 15% improvement with dSNRw technique). For N100 ERP, the improvements were not as dramatic (8% for traditional vs. 10% for dSNRw technique), and may be reflective of the inherent robustness of the N100 ERP [27]. In line with the effect size results, complimentary measurements including comparisons of ERP signal amplitudes and comparisons of differences among ERP experimental conditions further reinforced the superiority of the dSNRw approach (Figure 5 and Figure 6). ERPs generated using the dSNRw pooling resulted in larger ERP amplitudes and captured greater conditional differences, with improvements ranging from 8–12% compared to ERPs generated using traditional pooling.

Given that ERPs are generated as conditionally signal-averaged neural responses to pairs of stimuli (e.g., standard and deviant tone stimuli for P300) [7], increases in effect size implies a reduction of the variance of the difference among the two pairs of waveforms for the conditions, resulting in more robust capture of neural responses. The improved capture of neural responses is further confirmed by increased ERP signal amplitude and conditional differences. Most state-of-the-art ERP-based assessments currently rely upon the averaging of hundreds of trials [11,27], often resulting in long testing times, with 30–90 min being routine. However, as highlighted in previous work, most commonly used clinical assessments are pervasive in part due to their rapid assessment capabilities [36]. With improved effect size achieved using the dSNRw technique, there is now possibility to reduce the number of trials—and therefore test time—necessary to capture reliable neural signatures, thereby enabling more widespread applications of ERP-based techniques.

### 4.4. Caveats and Future Directions

While the results of the current study are promising for the initial validation of the dSNRw technique for signal augmentation, certain limitations of the study should be noted. As an initial study, the results of the dSNRw pooling technique were compared to results obtained from traditional channel pooling and single-electrode ERPs only within this study. However, this does not reflect an exhaustive comparative analysis with all possible signal combinatorial techniques (e.g., singular value decomposition or deep-learning based approaches). Furthermore, the analyses focused on specific electrodes, and future work should be undertaken for more widespread evaluation with other electrodes as well and also evaluating the topographical differences in the captured responses. Similarly, while beyond the scope of the current study, further work can be undertaken using simulated data to more comprehensively evaluate the impact of differing levels of noise among channels. Additional future directions include evaluation of the test/re-test reliability of ERPs generated using dSNRw combination and evaluation using consumer grade devices as well as comparisons with conventional ERP paradigms with large trial numbers. Finally, while this study utilized a sample size and best practices recommended for ERP studies [37], the results should be further verified using larger and/or distinct participant populations (e.g., patients) as well as other stimulation modalities (e.g., visual [38]).

### 4.5. Study Implications

ERP experiments focus on eliciting and assessing specific ERP features that are embedded within background EEG and other unrelated noise of neural, physiological, instrumentational, and environmental origin, which are often orders of magnitude greater than the ERP features of interest [7]. Signal conditioning and processing techniques such as differential amplification, filtering, trial averaging and channel pooling enable the isolation of the ERP features of interest by enhancing the contribution of the ERP features (‘signal’) and/or minimizing the impact of the unrelated artefactual noise [33]. However, techniques such as traditional channel pooling were developed in the era when most ERP experiments were undertaken within pristine laboratory environments, and EEG instrumentation mandated strict operating conditions within ideal parameter bounds (e.g., <5 kOhm skin-electrode impedances for all electrodes). In recent years, there has been a resurgence of interest in EEG, with experiments often undertaken outside laboratory environments (e.g., patient bedside [9,36], in-aircraft [39]), and modern EEG equipment now enables operations in less ideal conditions (e.g., <30 kOhm skin-electrode impedance with active electrodes [1]). As such, while the relative signal and noise contributions may have been similar across channels when experiments were undertaken within pristine environments and with lower impedances, that may no longer be the case when operating in more realistic environments and with higher skin-electrode impedances [18]. The dSNRw technique thereby brings the channel pooling approach into the modern era by explicitly assessing the SNR of the channels being pooled and enabling dynamic combinations in order to retain the maximum signal contributions and minimizing the noise influences.

## 5. Conclusions

In this study, we developed and evaluated a novel signal augmentation technique based on dynamically combining multi-sensor data through dynamic SNR-weighted pooling (dSNRw). Results from Monte-Carlo based simulations demonstrate the superiority of the dSNRw technique in capturing ERP effects of interest compared to the traditional channel combination approach in the realistic scenario of uneven noise levels among the channels being pooled. Real-world experimental data on healthy individuals using brain vital signs targeting the sensation (N100), attention (P300), and language processing (N400) ERP neural markers further confirmed the effectiveness of the dSNRw technique in improving the measured effect size of the ERP features of interest. With the increasing use of ERP-based techniques for monitoring across various brain diseases and disorders including traumatic brain injury and dementia, the improvements in ERP effect capture afforded by the dSNRw technique further optimize the translation of EEG capabilities from research settings into clinical and other out-of-laboratory applications.

## Data Availability

Data are not publicly available due to ethical approval constrains. However, requests for data that underlie the findings of this study can be made by contacting the corresponding author.

## Supplementary Materials

The following are available online at https://www.mdpi.com/article/10.3390/s21217258/s1, Figure S1: Comparison of Traditional and dSNRw pooling techniques at mastoid electrodes. The dSNRw technique captures significantly better ERP effects of interest compared to the traditional channel pooling technique across all three (N100, P300 and N400) ERP components. For each ERP component, values shown for 1000 permutations of effect size calculations as mean ± std. error. Diamond signifies *p* < 0.05 across pooling techniques.
