# Supplementary material for: Event Related Potential Signal Capture Can Be Enhanced through Dynamic SNR-Weighted Channel Pooling
Source: Sensors (Basel). 2021 Oct 31;21(21):7258. doi: 10.3390/s21217258 (PMC8588067; doi:10.3390/s21217258)
Supplement: Supplementary file 1 [file sensors-21-07258-s001.zip › sensors-1372180-supplementary.pdf]

Supplementary Materials: The following are available online at www.mdpi.com/xxx/s1,

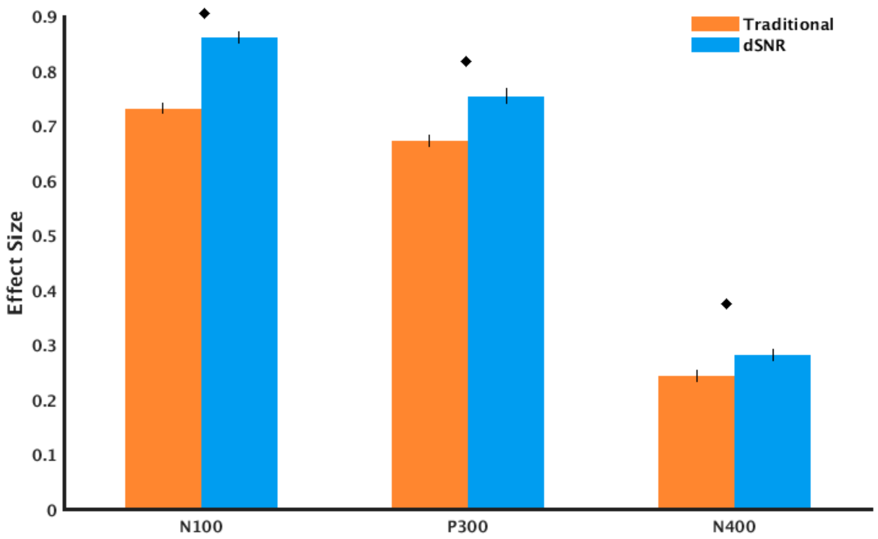

Figure S1. Comparison of Traditional and dSNRw pooling techniques at mastoid electrodes. The dSNRw technique captures significantly better ERP effects of interest compared to the traditional channel pooling technique across all three (N100, P300 and N400) ERP components. For each ERP component, values shown for 1000 permutations of effect size calculations as mean ± std. error. Diamond signifies $p < 0.05$ across pooling techniques.
